# Prosthetic push-off power in trans-tibial amputee level ground walking: A systematic review

**DOI:** 10.1371/journal.pone.0225032

**Published:** 2019-11-19

**Authors:** Roy Müller, Lisa Tronicke, Rainer Abel, Knut Lechler

**Affiliations:** 1 Department of Orthopedic Surgery, Klinikum Bayreuth GmbH, Bayreuth, Germany; 2 Institute of Sport Sciences, Friedrich Schiller University Jena, Jena, Germany; 3 R&D Össur, Reykjavik, Iceland; Holland Bloorview Kids Rehabilitation Hospital, CANADA

## Abstract

**Objective:**

Unilateral trans-tibial amputation signifies a challenge to locomotion. Prosthetic ankle-foot units are developed to mimic the missing biological system which adapts push-off power to walking speed in some new prosthetic ankle-foot designs. The first systematic review including the two factors aims to investigate push-off power differences among Solid Ankle Cushion Heel (SACH), Energy Storage And Return (ESAR) and Powered ankle-foot units (PWR) and their relation to walking speed.

**Data sources:**

A literature search was undertaken in the Web of Science, PubMed, IEEE xplore, and Google Scholar databases. The search term included: ampu* AND prosth* AND ankle-power AND push-off AND walking.

**Study appraisal and synthesis methods:**

Studies were included if they met the following criteria: unilateral trans-tibial amputees, lower limb prosthesis, reported analysis of ankle power during walking. Data extracted from the included studies were clinical population, type of the prosthetic ankle-foot units (SACH, ESAR, PWR), walking speed, and peak ankle power. Linear regression was used to determine whether the push-off power of different prosthetic ankle-foot units varied regarding walking speed. Push-off power of the different prosthetic ankle-foot units were compared using one-way between subjects’ ANOVAs with post hoc analysis, separately for slower and faster walking speeds.

**Results:**

474 publications were retrieved, 28 of which were eligible for inclusion. Correlations between walking speed and peak push-off power were found for ESAR (r = 0.568, p = 0.006) and PWR (r = 0.820, p = 0.000) but not for SACH (r = 0.267, p = 0.522). ESAR and PWR demonstrated significant differences in push-off power for slower and faster walking speeds (ESAR (p = 0.01) and PWR (p = 0.02)).

**Conclusion:**

Push-off power can be used as a selection criterion to differentiate ankle-foot units for prosthetic users and their bandwidth of walking speeds.

## Introduction

The total number of individuals with lower extremity amputations in the US is expected to increase up to 3.6 million by 2050 and it is estimated that 38% of all amputations will be major lower limb amputations [1]. As a result, the pursuit for an adequate replacement of the missing structures led to the development of a variety of different ankle-foot units covering and reflecting the manifold needs and applications given by the end users daily life/routines.

The positive power burst generated during the step-to-step transition is defined as push-off power [2] generated by the plantar flexors in non-amputee level walking. Push-off occurs during the first half of double-support stance phase and throughout the latter half of double-support stance when the weight-accepting foot is being lowered to the ground. In the final stage of push-off weight shifts to under the toes [3]. A weaker push-off results in a longer double-support stance time and is therefore, relevant to balance and dynamic stability [4] a specific challenge for individuals with lower limb amputation [5]. When walking at higher speeds, the ankle provides net positive work during push-off accounting for approximately half of the total mechanical work that is performed by the joints of the lower limb in the sagittal plane during a step [6]. Concerning the role of push-off work of the ankle, it has been debated whether it contributes primarily to leg swing or centre of mass (COM) acceleration. Zelik and Adamczyk [2] advocated that interpretations of ankle mechanics should abandon a contrasting view of leg swing versus COM acceleration and that ankle push-off should be perceived in a unified way with the effects being mutually consistent, thus underlining the importance of push-off power during human prosthetic locomotion.

In contrast to non-amputee subjects, unilateral lower-limb amputees exhibit reduced push-off which causes asymmetrical gait and increases load on the contra-lateral side [7–10]. The increased loading of the sound side is indicated by an increase of the magnitude of the first peak of the ground reaction force, the increased loading rate of the vertical ground reaction force in early stance and the first peak of the external knee adduction moment. These factors may accelerate the development of e.g. joint osteoathritis [11,12].

The evolution of prosthetic ankle-foot units has undergone impressive developments in recent decades, mainly focused on the generation of adequate push-off power in functional performance. State-of-the art Energy Storing And Return (ESAR) ankle-foot units, i.e. Ossur’s Flex-Foot and Seattle Light generated almost 3 respectively 2 times greater energy when compared to the early Solid Ankle Cushion Heel (SACH) ankle-foot units [13]. Other studies comparing these designs showed that increased push-off power contributed to a reduction of metabolic cost with a prototype of a controlled ESAR [14] and increased self-selected walking speeds with an advanced ESAR, i.e. Ossur’s Flex-Foot [15].

Further technological advancements include the powered (microprocessor controlled) ankle-foot unit (PWR) which is also associated with an increased peak ankle power [16,17] and a reduction in metabolic cost [17–19]. However, the relationship between metabolic energy and mechanical work is a complex one and the results of other studies have shown that powered ankle-foot units do not necessarily reduce metabolic cost, despite increased ankle push-off work [20].

In healthy non-amputee subjects, the level of ankle push-off power increases with walking speed [21]. Similar to non-amputee control subjects, unilateral lower-limb amputees exhibit increased push-off ankle power with increasing walking speed [22–24]. For example, when walking with the iWalk’s Power Foot BiOM (the only type of commercial powered prosthetic foot in the market at this time) peak ankle power increases from about 1.3 W/kg at 0.75 m/s walking speed to about 4.2 W/kg at 1.75 m/s [23]. However, corresponding experiments with ESAR or SACH ankle-foot units are not known to us. Moreover, there seems to be no studies comparing the push-off power of the three different ankle-foot units (SACH, ESAR, PWR) at various walking speeds.

Thus, the objective of this systematic review was to identify push-off power differences and their relation to walking speeds in three types (SACH, ESAR and PWR) of both pre-market (prototypes) and commercial ankle-foot units. The secondary objective was to evaluate if the push-off power differs among prosthetic ankle-foot types when walking speed increases.

## Materials and methods

This review was performed in compliance with the preferred reporting items for systematic review and meta-analysis protocols [25]. For the literature search the databases of Web of Science, PubMed, IEEE xplore and Google Scholar were accessed from the initiation of the databases up to June 17^th^, 2019. The used search term was: ‘ampu*’ AND ‘prosth*’ AND ‘ankle-power’ AND ‘push-off’ AND ‘walking’. Only journal papers and extended conference proceedings (e.g., IEEE) in the English language were considered.

**Fig 1:**
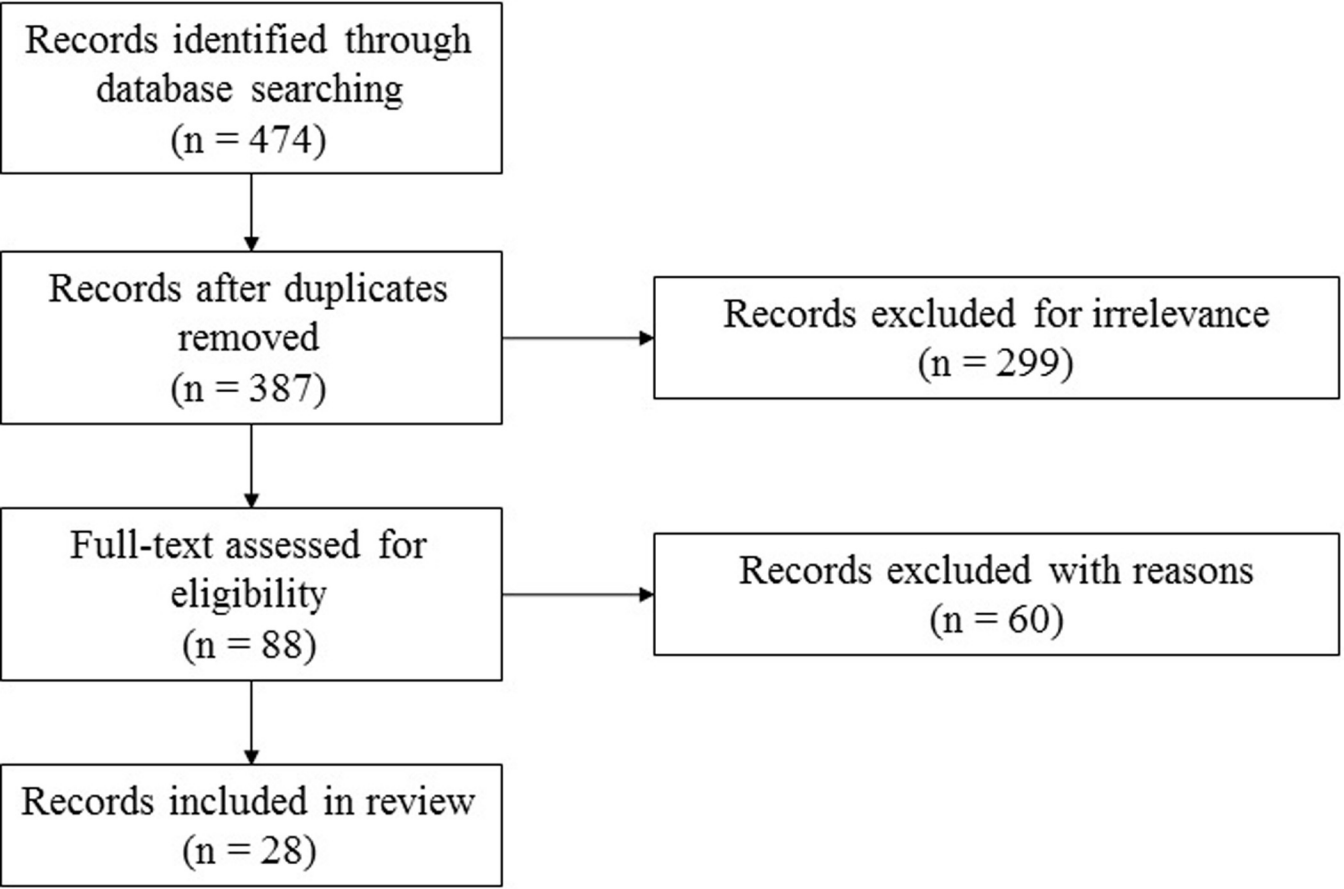
Flowchart showing the inclusion process.

After removing duplicates, two reviewers (R.M. and K.L.) independently reviewed the remaining publications for eligibility based on the title and abstract. The following topics led to the inclusion of a publication: 1) unilateral trans-tibial amputees with lower limb prosthesis and 2) reported analysis of ankle power during level walking (also case studies). Exclusion criteria were: 1) purely theoretical concepts, 2) robotics, 3) non-amputees with simulated amputation (e.g. wearing an ankle-foot prosthesis emulator), 4) non-amputees walking with an ankle-foot-orthosis or an ankle exoskeleton, 5) partial foot amputees, 6) pediatric amputees, 7) trans-femoral or bilateral trans-tibial amputees and 8) running or jumping. After the first screening of the title and abstract, full texts were obtained for publications considered potentially eligible. The flow diagram in Fig 1 depicts the review process.

Data extraction from the included publications was completed by one of two reviewers (R.M.), with a third reviewer (L.T.) checking the extracted data for accuracy and completeness. Any identified discrepancies were discussed by all three reviewers to ensure the accuracy of the extracted data.

The data extracted from the included studies were: 1) the clinical population examined (number of subjects, age, weight, height), 2) the type of the prosthetic ankle-foot units (SACH, ESAR, PWR; see below), 3) walking speed, 4) peak ankle power together with 5) walking terrain (treadmill or over ground) and the used calculation method.

The ISO standard (13405–2:2015(E)) provides a classification and description of prosthetic components related to their motion, activation and controls without any attempt to categorize performance levels [26]. However, identifying selection criteria for prosthetic ankle-foot units with regard to performance levels and intended use appears to be difficult because of confounding factors (users´condition, capacity and anthropometrics, prosthetic alignment, interface-fit). Thus, a multifactoral modelling approach would be underpowered because the sample sizes in the existing literature are too small. Cherelle et al. [26] selected prosthetic ankle-foot units based on the historical design development a) conventional feet (SACH), b) early (i.e. SAFE Foot), advanced (i.e. Ossur’s Flex-Foot) and articulated ESR feet (ESAR), and c) bionic feet (i.e. iWalk’s Power foot BiOM and several prototypes). Unlike Cherelle et al. [26] we assigned the articulated ESR feet (i.e. prototype of a controlled ESAR) as PWR because they make use of electronics and small servomotors to engage or disengage locking mechanisms.

Statistical analysis (SPSS 20; Chicago, IL, USA) was performed using linear regression to determine whether the ankle push-off power of different types (SACH, ESAR, and PWR) of pre-market (prototypes) and commercial ankle-foot units varied with regard to walking speed. Pearson correlation coefficients were used to determine the association between walking speed and the different types of the prosthetic ankle-foot units (SACH, ESAR, and PWR). To compare the push-off power of the different prosthetic ankle-foot units, a median split on walking speed was implemented.

Based on the median split, we employed two one-way between subjects ANOVAs (separately for slower and faster walking speeds) with Post-hoc analysis (Tukey HSD) to compare the push-off power between the prosthesis and non-amputated controls (NA). To compare the push-off power for slower and faster walking speed we performed a one-way ANOVA, separately for SACH, ESAR, PWR and NA. The significance level was set at p < 0.05.

## Results

The search retrieved a total of 474 publications across the four selected databases. 88 of them were classified as potentially eligible on preliminary screening. Assessment of full texts resulted in 31 publications meeting the quality criteria. In three cases, two publications appeared to be related to the same dataset (Fig 1). Thus, 28 publications were rated eligible for inclusion in the systematic literature review.

Of the 28 studies shown in Table 1, seven investigated the push-off power in SACH (earliest included study was published in 1993 [27]), 18 examined the push-off power in ESAR (earliest included study was published in 1995 [28]) and 14 the push-off power in PWR ankle-foot units (earliest included study was published in 2011 [29]). Three of the 28 studies compared SACH with ESAR [28,30,31] and eight publications compared ESAR with PWR. Comparison between non-amputees (NA) and different prosthesis were found in five publications, namely: one publication compared NA with SACH [27], two compared NA with ESAR [32,33], one compared NA with PWR [23], and one compared NA with ESAR and PWR ankle-foot prostheses [17]. None of the 28 studies compared all three different ankle-foot units.

**Table 1:** Studies included in systematic review.

Articles	N	Age [years]	Weight [kg]	Height [cm]	Prosthesis	Walking speed [m/s]	Peak power [W/kg]	Terrain	Methods
Realmuto et al., 2019 [37]	1	50	82	180	ESAR	1.0	0.71	treadmill	RFS
Heitzmann et al., 2018 [32]	11	40±12	81±17	180±9	ESAR	1.33–1.39	1.48–2.89	ground	UD
	11	37±11	76±12	179±8	NA	1.44	4.33	ground	
Houdijk^a^ et al., 2018 [44] or Wezenberger et al. 2014 [47]	15	56±11	86±13	174±4	SACH	1.22	0.6	ground	SA
					ESAR	1.22	1.7		
Childers & Takahashi, 2018 [39]	5	44±14	81±14	173±8	ESAR	1.1	0.75–1.1	treadmill	UD
Tahir^a^ et al., 2018 [22]	2	35±1	96±19	183±2	PWR ^d^	0.75–1.65	1.1–3.3	ground	RFS
Ray^c^ et al., 2018 [38]	22	53±12	102±19	178±8.	ESAR	0.85	1.0	ground	UD
Grimmer et al., 2017 [34]	1	17	55	-	ESAR	1.1	1.38	treadmill	other
					PWR ^g^	1.1	1.71		
Weinert-Aplin^c^ et al., 2017 [33]	10	28±4	90±14	182±5	ESAR	1.36	2.26	ground	RFS
	10	30±6	78±8	182±5	NA	1.29	3.12	ground	
Jeffers & Grabowski^a^, 2017 [64] or Montgomery & Grabowski, 2018 [56]	10	42±11	77±15	170±8	ESAR	1.25	2.05	treadmill	RFS
					PWR ^d^	1.25	2.05		
Feng & Wang, 2017 [24]	3	47±11	69±13	171±1	PWR ^h^	0.5–1.1	0.88–2.0	treadmill	other
Esposito et al., 2016 [17]	6	29±6	93±6	181±10	ESAR	1.24	1.9	ground	RFS
					PWR ^d^	1.24	2.95		
	6	23±5	91±12	179±11	NA	1.21	2.3	ground	
Quesada^a^ et al., 2016 [20]	6	47±6	88±9	179±4	PWR ^i^	1.25	2.95	treadmill	RFS
Huang^a^ et al., 2015 [41]	5	55±19	-	-	ESAR	1.0	1.35	treadmill	other
					PWR ^f^	1.0	3.1		
Doyle et al., 2014 [42]	10	36±8	88±18	176±7	ESAR	1.29	2.2	ground	ns
Huang et al., 2014 [35]	1	57	90	188	ESAR	1.0	1.25	treadmill	ns
					PWR ^f^	1.0	3.0		
Pickle^b^ et al., 2014 [65]	9	30±6	95±8	180±10	ESAR	1.25	1.48	ground	RFS
					PWR ^d^	1.25	3.29		
Zhu et al., 2014 [36]	1	-	70	-	PWR ^j^	1.25	3.3	ground	other
Grabowski & D’Andrea, 2013 [23]	7	45±6	100±10	181±8	PWR ^d^	0.75–1.75	1.3–4.2	treadmill	RFS
	7	48±7	98±12	186±6	NA	0.75–1.75	1.4–4.2	treadmill	
Hill & Herr, 2013 [48]	2	34±8	71±2	174±1	PWR ^d^	1.25	4.0	ground	RFS
De Asha^a^ et al., 2013 [66]	8	45±11	83±19	177±5	ESAR	0.93–1.38	1.2–1.05	ground	SA
Yeung^a^ et al., 2013 [30]	1	47	75	170	SACH	1.14	0.2	ground	ns
Segal et al., 2012 [46] or Morgenroth et al., 2011 [10]	7	52±12	81±10	185±5	ESAR	1.14	1.4	ground	SA
					PWR ^e^	1.14	3.2		
Ventura^a^ et al., 2011 [31]	12	49±17	82±13	178±6	SACH	1.2	0.4	ground	RFS
					ESAR	1.2	1.25		
Zelik^a^ et al., 2011 [29]	5	50±13	77±3	-	ESAR	1.14	1.36	ground	SA
					PWR ^e^	1.14	3.1		
Prince et al., 1998 [67]	5	42±14	80±2	175±7	SACH	1.15	0.65	ground	SA
Allard et al., 1995 [28]	1	24	-	-	SACH	1.21	0.99	ground	RFS
					ESAR	1.39	1.36		
Hubbard & McElroy, 1994 [43]	20	-	-	-	SACH	0.8	0.8	ground	RFS
Prince et al., 1993 [25]	6	20±6	61±10	168±7	SACH	1.49–1.84	0.5–1.01	ground	RFS
	5	19±8	58±15	166±13	NA	1.54–1.9	2.71–4.5	ground	

UD = UD model [50]; AS = segmentally-agnostic [40], no assumptions are made about the foot rigidity [49]; RFS = rigid foot segment model, foot assumed as rigid-segment; other = individual calculation performed, or different model applied; ns = foot model not specified.

^a^ Peak power values are estimated from published figures.

^b^ The author provided additional data not mentioned in the publication.

^c^ Missing parameters were retrieved from a previously published study using the same data set.

^d^ PWR = BIOM (iWalk, USA).

^e^ PWR = CESR (prototype, USA).

^f^ PWR = powered ankle-foot with pneumatic artificial muscles (prototype, USA).

^g^ PWR = Walk-Run Ankle (pre-market ankle-foot unit, SpringActive, USA).

^h^ PWR = PKU-RoboTPro-II (prototype, Peking University, China).

^i^ PWR = Powered ankle-foot prosthesis emulator (prototype, University of Pittsburgh, USA).

^j^ PWR = PANTOE (prototype, Peking University, China).

The sample sizes differed between publications and ranged from one [28,30,34–37] and 22 participants [38]. In total, 192 trans-tibial amputees were included in our statistical analysis.

The methods for calculating the push-off power varied between publications (Table 1): 13 publications reported to have made a rigid foot segment (RFS) assumption; three [32,38,39] used the UD model (UD) and the method in five publications is described as segmentally-agnostic (SA) [40]. Additionally, four studies [24,34,36,41] determined push-off power directly from parameters given by the powered device or applied individual calculations (other) and three studies [30,35,42] did not specify details of push-off power calculations.

**Fig 2:**
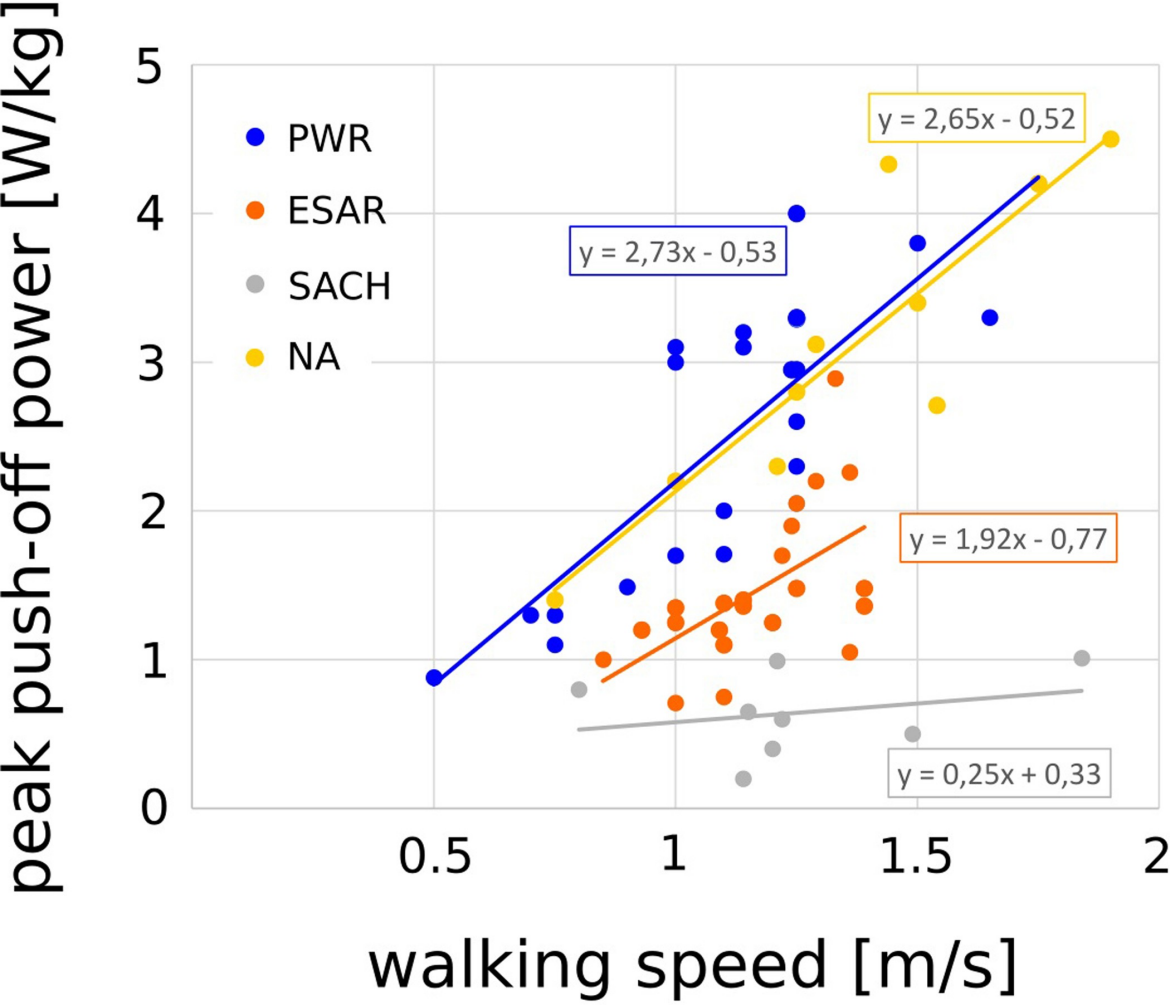
Linear regression between walking speed and peak ankle push-off power for different ankle-foot units and non-amputees. Regression lines represent the data of the included studies independent of the sample size. The equation of regression are given for different ankle-foot units (SACH, ESAR, and PWR) and non-amputees (NA).

The investigated walking speed ranged from 0.8 [43] to 1.84 m/s [27], from 0.85 [38] to 1.39 m/s [28,32] and from 0.5 [24] to 1.75 m/s [23] for the SACH, ESAR, and PWR ankle-foot units (Fig 2). Furthermore, the results of the peak ankle push-off power ranged from 0.2 [30] to 1.01 W/kg [27], from 0.71 [37] to 2.89 W/kg [32], and from 0.88 [24] to 4.2 W/kg [23] for the SACH, ESAR and PWR ankle-foot units (Fig 2). Positive correlations between walking speed and peak push-off power were found for ESAR (r = 0.568, p = 0.006) and PWR (r = 0.820, p = 0.000) but not for SACH (r = 0.267, p = 0.522).

**Fig 3:**
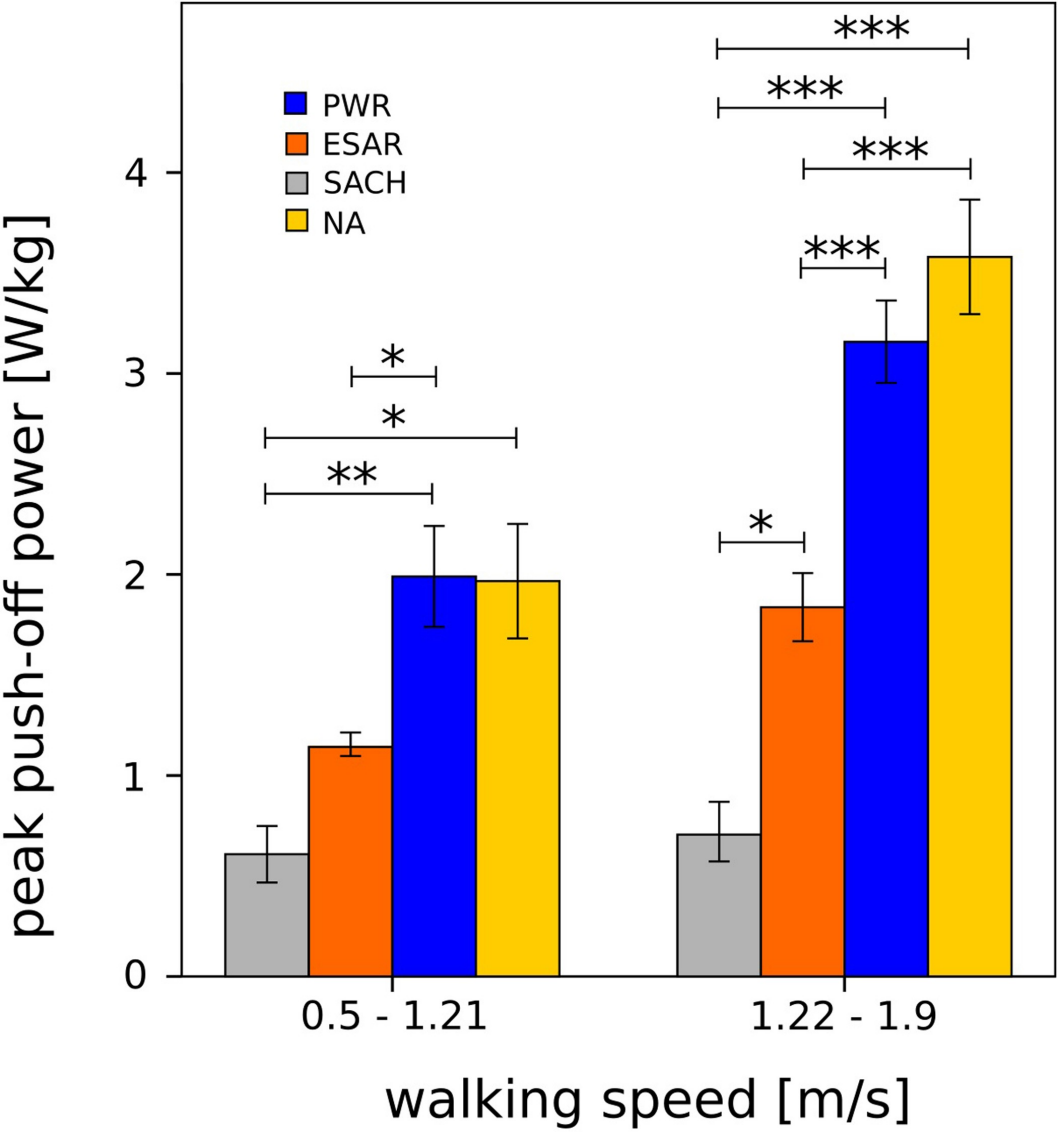
Peak ankle push-off power for different types of ankle-foot units and non-amputees. Mean scores of peak ankle push-off power between different types of ankle-foot units (SACH, ESAR, and PWR) and non-amputees (NA), separated for slower (0.5–1.21 m/s) and faster (1.22–1.9 m/s) walking speeds. Error bars represent standard errors. Significant differences are indicated with ‘*’ (p < 0.05), ‘**’ (p < 0.01), and ‘***’ (p < 0.001), respectively.

For slower walking speeds, significant differences in the push-off power were established between SACH (0.61 ± 0.31 W/kg) and PWR (1.99 ± 0.87 W/kg), SACH and NA (1.97 ± 0.49 W/kg), and ESAR (1.16 ± 0.23 W/kg) and PWR (Fig 3). For faster walking speeds, significant differences in the push-off power were found between SACH (0.70 ± 0.27 W/kg) and ESAR (1.84 ± 0.54 W/kg), SACH and PWR (3.16 ± 0.68 W/kg), SACH and NA (3.58 ± 0.75 W/kg), ESAR and PWR, and ESAR and NA (Fig 3). Additionally, when comparing the push-off power for slower and faster walking speeds significant differences were identified for ESAR (p = 0.01), PWR (p = 0.02), and NA (p = 0.01).

## Discussion

To our knowledge, this paper is the first to compare the push-off power of three different kinds of prosthetic ankle-foot units (SACH, ESAR, and PWR) at different walking speeds. The results of our analyses demonstrated that a positive correlation between walking speed and push-off power can be found when walking with the PWR or ESAR but not for SACH feet. Furthermore, we found that for higher walking speeds (> 1.21 m/s), push-off power performances among the different types increased whereas at lower walking speed (≤ 1.21 m/s) the differences in push-off power performance were lower. Thus, push-off power can be used as a selection criterion to differentiate between ankle-foot units for prosthetic users with regard to their bandwidth of walking speeds.

### Different levels in push-off power

With the objective to compare the push-off power of three different kinds of prosthetic ankle-foot units (SACH, ESAR, and PWR) it was demonstrated that their push-off power performance progresses differently at different walking speeds. In able-bodied individuals positive peak ankle push-off power increases with walking speed [21]. To mimic human gait, PWR ankle-foot units adjust push-off power based on walking speed, i.e. push-off power increases with walking speed [22–24]. This has already been proven in the literature and can also been seen in Fig 2 (similar slopes of the PWR and NA regression lines) and Fig 3 (no significant differences of peak push-off power for slower and faster walking speeds).

A positive correlation between walking speed and push-off power can also be found when walking with the ESAR (Fig 2), but with a flatter slope of the regression line when compared to the PWR (PWR slope: 2.73, ESAR slope: 1.92). With the SACH ankle-foot unit, the push-off power does not increase with walking speed (Fig 2). Shorter step length, lower gait symmetry [44], increased sound limb loading [15], increased energy cost, reduced gait efficiency in declines and inclines at higher speeds [45] are the results in comparisons when push-off power increased in prosthetic ankle-units.

The differences in push-off power between the prosthetic ankle-foot units became larger with increased walking speed (Figs 2 and 3). For slower walking speeds (< 1.22 m/s), significant differences in the push-off power were present between SACH and PWR, and ESAR and PWR (Fig 3). No significant differences were found between SACH and ESAR. For faster walking speeds, significant differences in the push-off power can be found between SACH and ESAR, SACH and PWR, and ESAR and PWR (Fig 3). Thus, the benefit of push-off power in the PWR compared to ESAR and SACH, and respectively ESAR to SACH, was more pronounced at faster walking speeds.

### Prosthetic ankle-foot designs

During push-off the ESAR ankle-units deliver less power than the PWR [10,17,29,46] but more power than the SACH [28,31,44,47]. SACH ankle-units are composed out of a solid core and resilient material. Their dynamics rely on the recoil of the resilient material both in the heel and the toe. A typical ESAR foot with a direct bolt-on heel to toe blade stores energy within the composite material by deformation during early to mid-stance and then allows those elastic structures to recoil and release the energy to provide energy for propulsion contributing to push-off during pre-swing. Within the ESAR, the Pro-Flex showed distinct push-off power performance (Table 1 and Fig 2). The Pro-Flex delivered similar peak ankle power during push-off as the PWR ankle-foot units and more energy during push-off when compared to the Vari-Flex (ESAR) ankle-unit [32]. The 3 blade-linkage system on the Pro-Flex Foot is designed to pivot the shank section faster over the foot section and increases progressively the stiffness at terminal stance. In consequence the forefoot section is loaded more rapidly at initial stance and allows for more prosthetic foot dorsiflexion at late stance [39]. More dorsiflexion results in more energy stored in the forefoot section which increases the energy returned during push-off. The PWR add external power generation (e.g. by using a serial elastic actuator [48]) into the kinetic chain and thereby increase the potential energy available at pre-swing.

### Push-off power modelling approaches

The approaches for calculating the push-off power vary between studies. In most studies the data for push-off power calculations were collected via 3D motion analysis. The conventional models were based on the assumptions that the foot is a rigid body segment (RFS) and has a defined joint articulation point. Both assumptions are violated when measuring prosthetic feet with flexible keels and no articulation point [40,49]. The method of Prince et al. [49] defines net prosthetic power as sum of rotational and translational power. The necessary parameters are determined at a rigid part at the distal end of the prosthetic leg. The method does not rely on the previously mentioned assumptions and can be described as segmentally-agnostic [40], neither does the UD model which provides an estimate for below-knee structures but without determining an ankle joint center or foot segment [50]. The model combines rigid and deformable model components. Zelik et al. [40] showed that the conventional 3 DOF ankle power, defining the foot as single rigid body, tends to overestimate the net power compared to ankle foot power calculations without a single rigid body assumption or models that relax this assumption by a different segmentation of the foot. Besides the used marker model, marker placement and shoes impact the measurements of push-off power [51,52]. Due to the wide variance in shoe characteristics, shoe wear is hardly controllable and has therefore not been considered within the underlying review. Same holds true for marker placement, e.g. the position of the ankle marker from the Plug-in Gait model may be difficult to define precisely as no obvious joint center may exist depending on the foot design and is usually subject to individual estimation of the tester. The significance of the selected measurement methodology should be considered when examining ankle power results of prosthetic feet.

### Clinical outcomes

Key factors limiting the ability of lower limb prosthetic users are associated with increased risk of falls, risk of osteoarthritis, occurrence of lower back pain and skin injuries [53–56]. The influence of push-off power has not been linked to risks within this literature review.

Gait after unilateral lower limb amputation is reported to be asymmetrical and it is thought that the asymmetrical limb loading may lead to the majority of the above-mentioned limitations. Within the included publications, the definition of symmetry was based on many different parameters. Step length symmetry was reported to improve when comparing SACH versus ESAR [44]. However, during level walking PWR and ESAR provided similar symmetry results [57].

Grabowski et al. found a reduction of 20% in external knee adduction moment (EAM) on the sound side when using a powered ankle-foot prosthesis [23]. Morgenroth et al. reported similar results, namely, that EAM was reduced by 26% on the sound side when using a PWR compared to a conventional ankle-foot unit (i.e. Seattle Light 2). Even though the ankle push-off power of the PWR was significantly higher than ESAR, differences in EAM were not statistically significant between these two foot types [10]. In literature an increased EAM is believed to contribute to the development of osteoarthritis [58,59].

Additionally, metabolic cost has been frequently investigated in relation to prosthetic push-off power but results are inconclusive. Five publications [17,19,60–62] report on reduced metabolic cost resulting from the presence of active push-off in prosthetic foot-ankle units however, these publications did not report the push-off power values. In contrast, three publications included in the present systematic review reported that increased push-off power work did not reduce metabolic cost in trans-tibial amputees during level walking [20,46,57].

In summary, none of the publications included in this paper established a direct link between increased push-off power and clinical outcomes. More research is needed in the future to link the specific performance of a device to a particular clinical outcome due to the fact that confounding factors are manifold and cannot be excluded. Typical limitations concerned with prosthetic research studies is the absence of clinically authoritative differences due to the sensitivity of the outcome measures and their sparse validation. However, future research is needed to reveal whether physiological ankle push-off power has a positive effect to mitigate comorbidities such as osteoarthritis or lower back pain. Future research might observe prosthetic users during their daily routine (e.g. when descending curbs [63]) in order to gain more insightful knowledge regarding potential clinical benefits of push-off power.

## Competing interests statement

The authors have read the journal's policy and the authors of this manuscript have the following competing interests: R.M. and R.A. are employees of Klinikum Bayreuth GmbH and L.T. and K.L. are employees of Ossur hf. a medical device manufacturer who produces some of the products included in this review. This does not alter adherence to PLOS ONE policies on sharing data and materials. There are no patents, products in development or marketed products associated with this research to declare.

## Supporting information

S1 Checklist (DOC)
